# Analytics in sustainable precision animal nutrition

**DOI:** 10.1093/af/vfz003

**Published:** 2019-04-11

**Authors:** Douglas M Liebe, Robin R White

**Affiliations:** Department of Animal and Poultry Sciences, Virginia Tech, Blacksburg, VA

**Keywords:** computer vision, data mining, Internet of things, machine learning

**Implications**

- The global population, resource, and climate dynamics suggest we must improve sustainability of food production systems; precision feeding of livestock may be one way to accomplish this goal.
- Analytics for precision management can be classified according to four levels: I) technique, II) data interpretation, III) integration of information, and IV) decision making. Most current animal agricultural analytics fall under categories I and II. Moving toward analytics that address integration of information and decision making is of critical importance.
- Data analytical techniques such as linear modeling and machine learning provide unique and important tools for interpreting data obtained from on-farm sensors. These techniques each apply to the different levels of precision management classification.
- Assessing adequacy and performance of analytics tools must, by default, depend on the objective of those tools and the type of response considered. As more advanced level III and IV systems are developed, integration of expert opinion into analytics may be essential to optimize performance and relevance on-farm.

## Introduction

**Figure 1.**
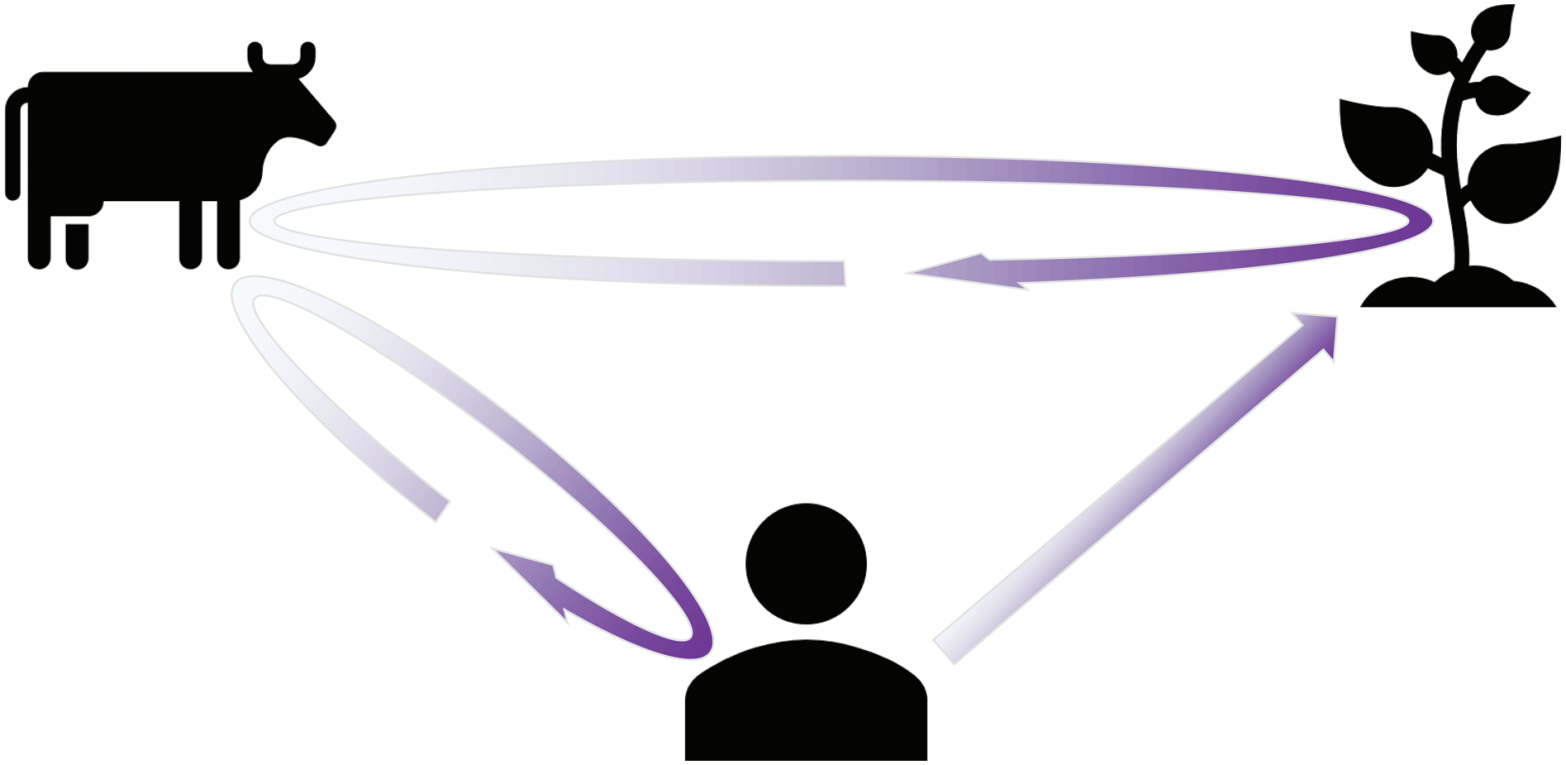
Depiction of the feedback loops between the farm manager, animal, and environment. The animal and environment influence each other, as do the animal and the manager’s decisions about the animal. Additionally, the manager can make decisions about the environment that will influence the animal.

The global population, resource, and climate dynamics suggest we must improve sustainability of food production systems (Ohlsson, 2014; Kleinman et al., 2018). Improving livestock production sustainability is particularly important because a significant portion of the projected increases in global food demand is anticipated to come from livestock (Thornton, 2010). Improving sustainability of livestock production systems can be achieved through optimized reproductive, genetic, nutritional, and health management (White et al., 2014, 2015). Management decisions within livestock production can be thought of as two interleaved feedback loops. The first feedback loop is between the animal and the environment: the animal is influenced by its environment and, in turn, influences its environment. The second feedback loop is between the animal and the manager: the manager takes information about the animal’s behavior and attempts to influence the environment to optimize the animal’s performance (Figure 1). Managers make management decisions on different timescales ranging from immediate to relaxed. An example of an immediate management decision would be a farmer identifying an animal as sick, isolating the animal, and treating the animal for the illness. We term this immediate because the farmer must identify the sick animal as soon as possible and must react to the diagnosis as soon as possible. An example of a relaxed management decision would be the farmer electing to change the feed provided to his animals in response to something observed about their production (i.e., the cows are producing poorly, so change the ration to provide higher nutrient density to correct a nutrient shortfall). This decision is more relaxed because its formulation and response are subjected to natural, biological delays (i.e., it may take days to weeks to see a production response to a new diet). Improving the precision of these decision-making processes and reducing the burden of decision making on farmers are two critical steps toward improving sustainability of livestock production. Precision agricultural technologies have been identified as one possible solution (Berckmans, 2014; Tullo et al., 2019).

Precision field crop agriculture has dramatically expanded and industrialized over the last several decades, demonstrating substantial opportunity for using precision technologies in agriculture (Thorp and Tian, 2004; Nash et al., 2009; Zhang and Kovacs, 2012). Such technologies include global positioning system (GPS) guided equipment, unmanned aerial vehicles, robotic harvesting and monitoring equipment, automated application of agrochemicals, and others. Precision animal agriculture, on the other hand, has had limited expansion. Although technologies, such as temperature monitors, rumen sensors, robotic milking machines, and others exist, the uptake and industrialization of precision animal agriculture has not paralleled crop agriculture. There are several differences between crop and livestock management that may contribute to this difference in technology uptake. For example, the management time scales for crop agriculture interventions, while highly profitable, are often measured in days or weeks. In animal agriculture, timescales for certain management can range from hours to days. For issues of nutrition, health, productivity, and efficiency, animal agriculture must treat both the individuals and the collective, whereas crop agriculture focuses primarily on the field (rather than on individual plants). Animal losses are also perceived differently than crop losses, possibly imposing higher standards on animal-based decision technology. Collectively, these challenges mean that animal agriculture will likely require different types of technological interventions than have been pioneered in crop systems. Exploring opportunities for where precision technologies may be relevant in the livestock nutrition space exemplifies this need.

### Management applications for precision animal nutrition

#### Optimizing rumen fermentation.

 The idea that fermentation can be optimized if degradable carbohydrate sources and degradable protein sources are properly matched has been contemplated for decades (Sinclair, 1995). The theory behind optimizing nutrient synchrony suggests that fermentations will be optimized if they are never limited by energy or nitrogen (i.e., supplies are balanced). Despite this theory being sound, achieving nutrient synchrony within rumen fermentations is extremely difficult to accomplish with currently available technologies (Hall and Huntington, 2008). One potential reason for this challenge is the limited real-time data available on the fermentation environment. Several models attempt to account for nutrient degradation kinetics (Hanigan et al., 2013; Higgs et al., 2015; Van Amburgh et al., 2015; Li et al., 2018); however, obtaining data to construct and evaluate models of degradation kinetics in vivo often requires expensive experiments. The advent of technologies such as indwelling rumen sensors have enabled more precise understanding of how pH changes over the course of a day. Expanding these sensors to include recording other important metabolites could enable development of feeding recommendations that take fermentation profile into account more precisely.

#### Detection of metabolic diseases. 

It is possible to use analytics to identify risk of metabolic diseases. Existing efforts to identify other disease states (e.g., mastitis) have shown moderate promise. Much like metabolic diseases, mastitis is extremely costly to the dairy industry. Diseases are often difficult to predict due to the imbalance of positive results (disease cases) relative to the population. For example, the incidence rate of clinical mastitis ranges among farms and depends on many factors like housing or location. The national average is near 15 cases per 100 cow lactations, or 1 case per 2,033 cow days, assuming a 305 day lactation (McDougall et al., 2007). Put another way, a priori, a randomly selected lactating cow from a random herd is only approximately 0.05% likely to exhibit clinical mastitis. In some farms, this rate may be 0.1% or higher. The low density of the positive test cases and the variation in the expected rate of positive test cases both cause challenges for developing robust predictions.

Sparse datasets, the analytical term for the issue of having a disproportionate amount of positive test cases in a dataset, are a common problem in present-day analytics (Han et al., 2015; Greenland et al., 2016). However, due to the widespread nature of the issue, new analytical techniques such as modified tree-based algorithms can learn patterns while maintaining the underlying proportion of cases in the training data (Ushikubo et al., 2017). Alternatively, the collation of larger datasets is also advantageous for producing better metabolic disease predictions. There is a tendency to collect new data to train new models, but in cases with sparse data, the combination of past data and new data will lead to richer training sets. Consider that each additional positive training case will greatly improve accuracy compared with each new negative case. In fact, removing additional negative cases to artificially improve the proportion of positive cases can help to train models. The caveat to training on stratified datasets is that they must be properly validated on datasets with the appropriate proportion of positive cases to determine real-world use. By utilizing strategies designed for the problem of sparse data in machine learning, predicting metabolic disease will become easier, and most importantly, more accurate, providing decreased false-positives.

#### Response-based nutrient requirement recommendations.

 A major limitation of existing nutrient requirement systems like the National Research Council Requirements for dairy cattle (NRC, 2001) is the requirement-based nature of the recommendations. Maximizing production mass is often not the same as optimizing production efficiency. Multicriteria optimization has previously been used to formulate rations to simultaneously achieve multiple environmental goals (White et al., 2014, 2015). Optimizing productivity or economic parameters could also be accomplished with this technique if the underlying equations linked dietary inputs with productive outputs in a responsive way. A challenge with response-based nutrient requirements systems is that most of our current data that could be used to develop such a system relies on pen-fed cattle. Responses of individuals are likely unique and such a response-based model would be more useful if feeding systems and nutrition models did a better job of representing the individual, rather than the collective.

#### Precision nutrition research.

 In a wide variety of ruminant nutrition research, access to the rumen is obtained through rumen cannulae; however, sampling through this orifice is physically difficult and often results in mixing of naturally stratified (vertical and horizontal) rumen contents. The physical difficulty in sampling the rumen can impede precision monitoring of difficult-to-reach areas. Additionally, disrupting the rumen environment through sampling physically or chemically alters the unique microclimates that are thought to exist within the rumen, and thus precluding accurate and representative sampling. Collectively, these challenges make accessing unique microclimates within the rumen a challenge. The availability of a platform that can monitor rumen sensors would be valuable to the study of these unique rumen microclimates.

#### What limitations exist for current technologies?

 Rutten and colleagues summarized 126 publications describing 139 dairy sensor systems from the period 2002 to 2012 (Rutten et al., 2013). The systems were then compared based on the four levels of I) technique, II) data interpretation, III) integration of information, and IV) decision making. Systems that accomplish all four of these levels are often referred to as cyberphysical systems. These cyberphysical systems are often an automated network of sensors, networking technologies, analytics, and actuation technologies that work in combination with or independent of the farmer to affect management changes based on real-time sensed information on-farm. None of the 139 sensor systems evaluated by Rutten and colleagues included integration of sensed metrics with other information available on the farm to produce management advice or automated decision making (Rutten et al., 2013). Most sensor systems that were used in the farmer’s decision process only provided the raw data measured by the sensor, or a probability (such as the probability of disease given the sensor data). In both cases, the farmer is left to their intuition to integrate and actually make a management decision. Although basic linear models or logit models produce predictions that are correct on average over a group, these models cannot account for increased variation in individuals. The models being used to interpret data, as referenced in level II of Rutten et al. (2013) can struggle under the complexity of decision making. For example, although there may be a manageable number of factors that affect the prediction of ketosis, the number of factors affecting the costs and benefits of the treatment of said ketosis is surely greater. Put another way, knowing that a cow is 35% ± 2 likely to be ketotic tomorrow does not say anything about whether the farmer should check the cow, treat the cow, cull her, or something else. To properly assess the promise of analytics in creating cyberphysical systems capable of filling all four levels of the Rutten et al. (2013) summary of agricultural systems, we will present a common precision nutrition aim: automated individualized feeding of dairy cows. Using this example objective, we highlight several possible alternative analytical approaches and discuss their strengths and potential pitfalls relevant to this objective.

### A nutrition analytics example: automated individual feeding

#### Automated individualized feeding. 

Given the variation among individual animals, it is reasonable to assume that by using data specific to each animal, we can make better decisions on what, and how much, to feed. As we have previously noted, model-based feeding can optimize productivity for the whole farm because individuals likely have differing and unique requirements. Individual feeding requires the ability to collect data specific to each animal and analytics capable of estimating individual requirements from that data. Feeding individuals eliminates the need to over-feed some animals to avoid under-feeding others, likely leading to more targeted feeding practices. One does not necessarily need to feed each animal individually; this same reduction in over/under feeding can be accomplished simply by reducing the variation in the feeding group, either by feeding more like-animals together or by feeding animals in small groups. An example of variance reduction through smaller groupings of animals would be the use of different feeding groups by lactation number in dairy cows. It is clear that nutrient requirements are vastly different for first and fourth lactation cows, so they are separated to reduce the feed requirement prediction variance. Another more targeted example of individualized feeding is concentrate supplement feeding. A larger group of animals can receive the same basal diet and the supplement is provided separately to smaller groups (Dela Rue and Eastwood, 2017). However, this type of individualized feeding, as noted by Dela Rue and Eastwood (2017), has not been shown to provide marginal benefit to farmers. Multiple recent studies which suggested individualized supplement feeding saw no improvement in milk production, body condition score, or body weight (Lawrence et al., 2015; Dale et al., 2016; Little et al., 2016). Although it seems intuitive that more individualized feeding regimens would lead to better performance, this is not always what occurs in practice. These limitations may be because of the aforementioned issues with requirement models, which are based on data from groups of animals, not individuals. Another limitation might be the complexity of analytics used for feeding recommendations. Of the three citations above that showed no increase in performance on individualized concentrate feeding, all studies used only one variable (milk yield) to inform concentrate requirement. In one study, only two levels of concentrate based on milk yield were fed. In the other two studies, a linear multiplier of milk yield was used to determine concentrate. Such low-dimensionality models, using only one variable to predict a response, limits the robustness of the predictions and results. We will examine potentials of higher-level modeling approaches by examining the current infrastructure to support cyberphysical systems in the four levels described by Rutten et al. (2013).

#### Current cyberphysical systems infrastructure.

 Level I, the techniques for data collection, is comprised of technologies such as radio frequency identification (RFID) tags, accelerometers, and other output measurement software (e.g., inline milking parlor sensors). We can use these data that are collected daily, or even in real-time, to broadly evaluate the performance of animals. One of the issues with the techniques of collecting raw data is the interpretation. With only raw data, it is hard to determine the cause–effect relationship between feeding and performance. For example, the fact that the daily step count of an animal has increased on a new diet does not inform the farmer whether or not to continue feeding this diet or what needs to be changed. Rather, raw data must be interpreted before it can be used effectively to make diet decisions. Level II, or the interpretation of sensor data, seeks to add context to sensor data with emphasis on explaining such relationships. Many models attempt to predict intake requirements of dairy cows using raw data as predictors (Jensen et al., 2015). Jensen et al. (2015) evaluated models that were used on a national scale in different countries. All models were fit to held-out intake data to determine the residual error in each prediction model. The root mean square prediction error for each model ranged between 1.2 kg dry matter per day and 3.2 kg dry matter per day (Jensen et al., 2015). The held-out data included 94 treatment means derived from 917 lactating dairy cows. A given model’s average prediction was near 2.0 kg of dry matter greater or less than a cow’s average intake. If these results were applied to individual cow days, the variance would necessarily be greater than the variance in predictions for a cow’s average intake. Models predicting dry matter intake can be simple, lending themselves to being correct on average, which is not as useful in individualized feeding because response variance increases. In a review of linear models predicting dry matter intake (Jensen et al., 2015), models referred to as “advanced” were those that incorporated interaction terms into the linear model, specifically the models “TDMI” and “NorFor” (Huhtanen et al., 2011; Volden et al., 2011). Many recent publications involve predicting intake using less than 10 total predictor variables and rely on basic linear regression (McParland et al., 2014; de Haas et al., 2015; Shetty et al., 2017; White et al., 2017). Most models attempt to find the few variables that will reduce the variance better than previous models. At some point, we will not be able to find a selection of 10 or fewer variables that continue to reduce variance in a meaningful way. One advance in data analytics is hierarchical modeling, which works well in the case where there are many models using varying parameters to predict the same response. Making a “model of models” can improve accuracy beyond that of any one model in the group (Gelman, 2006). This is possible due to uncorrelated error structures in different submodels. To create an example hierarchical model for predicting dry matter intake in dairy cows, we could combine the outputs of models built on herd level data into models built on models using different individual cow measurements to make a more accurate prediction of individual dry matter intake than using a single model alone. Although hierarchical modeling is just a framework, there are many useful ways to combine existing models that can improve model accuracy. Models can be weighted based on accuracy in a test dataset, the variance of predictions, or even on prior knowledge.

**Figure 2.**
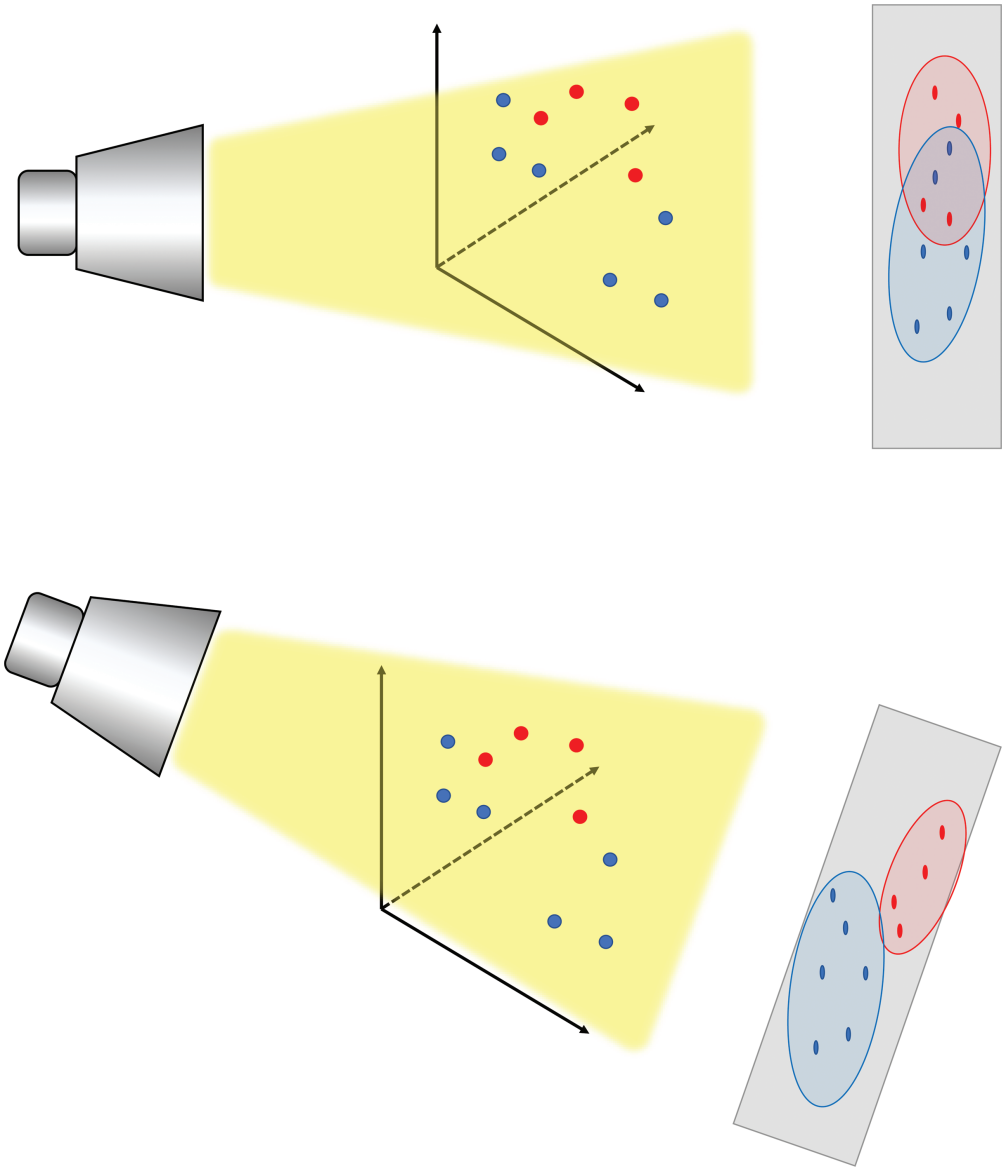
Example of principal component analysis from three to two dimensions. Consider flashing a light on a set of points in three dimensions and observing the shadows of the points in two dimensions on the wall. The shining of the flashlight through the data represents the search for the plane which creates the greatest variance between groups in the data. The angle of the light in the bottom picture finds a better two-dimensional plane to project the points onto compared with the image above.

**Figure 3.**
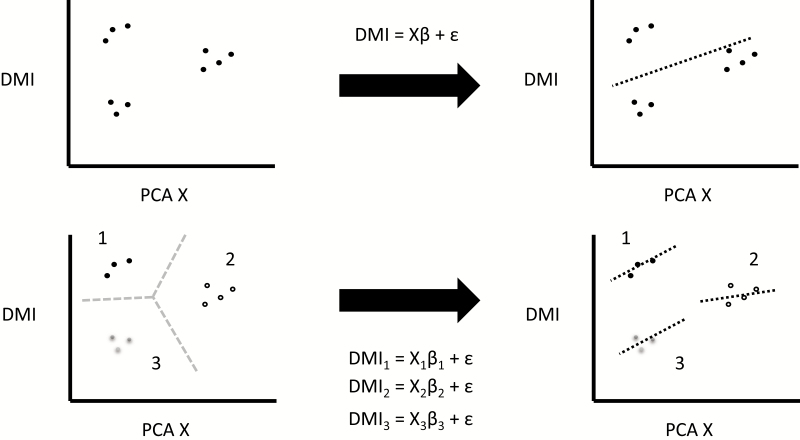
Comparison of fitting models after grouping results from principal component analysis (PCA). Grouping data based on a clustering algorithm allows the same model increased flexibility when making predictions. Notice that the model used does not change, only the data used to train the model is varied. DMI, dry matter intake.

With over 9 million dairy cows in the United States, it intuitively seems easy to collect sufficient data to predict intake; however, this is not necessarily the case (McParland et al., 2014). First, data must be collated, not dispersed, to create better-trained models. There are incentives now for farmers to continue to collect individual intake data and genetic data relating to intake to help inform farmers in the future (Berry et al., 2014). An estimated 89% of genetic variation in dry matter intake could be explained with only four common animal characteristics, according to one meta-analysis of genetic studies (Berry and Crowley, 2013). Although we have great amounts of data, there are near-infinite permutations of cow characteristics that would need to be predicted to improve dry matter intake prediction. Luckily, data analytics offers a way to reduce the dimensionality of problems and also group similar animals together to make the prediction space more manageable. Principal component analysis attempts to reduce dimensionality while maintaining maximal variance in the remaining dimensions using an orthogonal transformation (Pearson, 1901). Consider a three-dimensional set of data, shown in Figure 2. If we know the groupings ahead of time, we can find two angles using all three factors that maximizes variance in the dataset. This is modeled using a flashlight at different angles and shining it through the data and observing the shadow cast along the “wall’s” two axes. The angle of the flashlight that casts the shadow with the least variance within groups indicates the two planes to condense the data onto. By using all three factors but condensing the descriptors into two values for each point, we have reduced the dimensionality at minimal variance cost between groups. This is evident in the second image in Figure 2. Using principal component analysis can also help discern groups, as this analysis is sensitive to scale changes and can be used to determine the distance between two multi-dimensional points in space. Traditionally, a machine learning technique like *k*-nearest neighbors (Altman, 1992) or *k*-means (Lloyd, 1982) is used to determine the similarity between points. In our example with a herd of cows that we need to predict and feed individually, a linear model trained on the entire herd will only be right on average. If we do not have sufficient data to make low-variance predictions for individual cows, we could employ principal component analysis on the individual cow data to determine cows that are most similar, combine their data and train models on these smaller combined datasets of similar cows to achieve more accurate results. By using a fixed modeling procedure and measure of accuracy, we could iteratively test models using data from smaller groups until we no longer saw an improvement in accuracy. Consider the scenario outlined in Figure 3 which explains the framework for using principal component analysis to find the optimal groupings for a given model.

**Figure 4.**
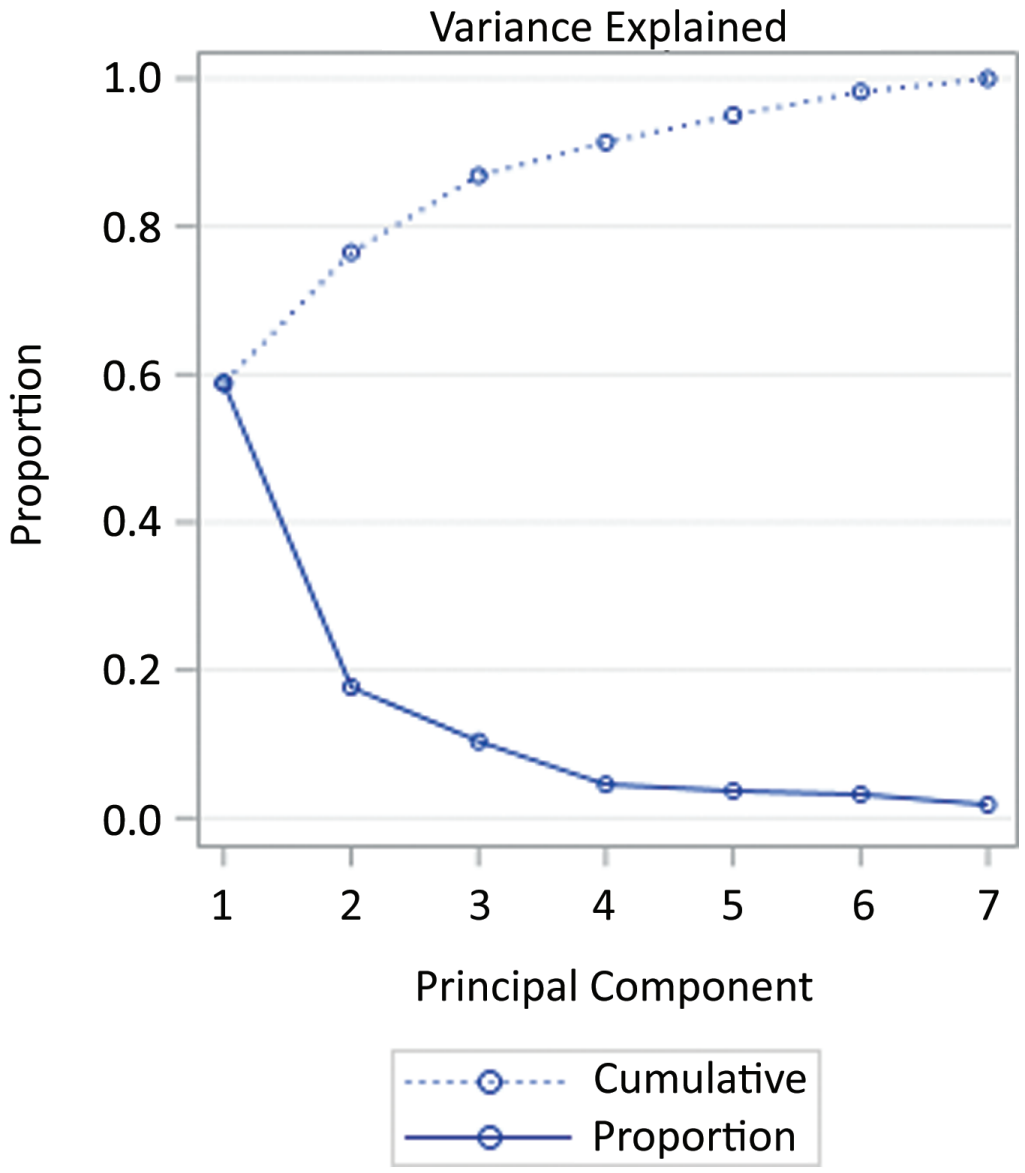
An example plot of the proportion of variance explained by each additional component in principal component analysis. Variance explained by each additional component can vary considerably based on the data you are working with (Shah et al., 2018).

It is important to note that although two-dimensional principal component analysis is easiest to visualize, these results should be retained in the number of dimensions that explains a specified amount of variance. Figure 4 shows a plot of the variance explained as the number of dimensions included in principal component analysis is increased. With fewer dimensions there is less variance explained by the components and the proportion of variance explained by each additional component is high. As we increase dimensions, the cumulative variance explained increases but the proportion of variance explained by each additional component decreases. Humans tend to interpret best in two dimensions, but we can see that if we wanted our principal component analysis to explain at least 80% of the variance in our dataset, two dimensions would not be sufficient. Also keep in mind that not all datasets will produce such steady reductions in variance with each component. There is no rule of thumb for how many components to condense. With principal component analysis, and many algorithms in data analytics, we must trade-off interpretability for accuracy.

### Opportunities to leverage machine learning in precision livestock nutrition

In level III, integration of information, the predictions made by models are used to created recommendations for the farmer. Level IV is the culmination of the prediction, leading to action, either by the system itself or the farmer. A lack of level III and IV cyberphysical systems was noted in Rutten et al. (2013). We would expect that, by utilizing the most appropriate modeling techniques to generate predictions at levels I and II, appropriate decision-making models would be possible. However, this is obviously not the case, as we see minimal examples of decision-making algorithms present in the current animal nutrition literature. One factor that traditional modeling frameworks do not allow for is the ability to update based on feedback. If a level II model predicts dry matter intake at 50 kg, but the farmer continuously adjusts this to 45 kg, based on his/her knowledge of something outside the model scope, a traditional model does not “learn.” Here, neural networks and other recurrent machine learning algorithms provide a promising approach to decision-making frameworks by allowing for revising predictions in practice. In a traditional individualized feeding modeling framework, a model is built for each cow and the model itself does not change, only the predictions. In a machine learning framework, the predicted dry matter intake for a cow each day could be predicted and, using all data available along with the actual response of the animal, the algorithm may change the weights of certain factors in the model. This dynamic feedback loop allows the model to “learn” on-farm and produce more accurate predictions.

**Figure 5.**
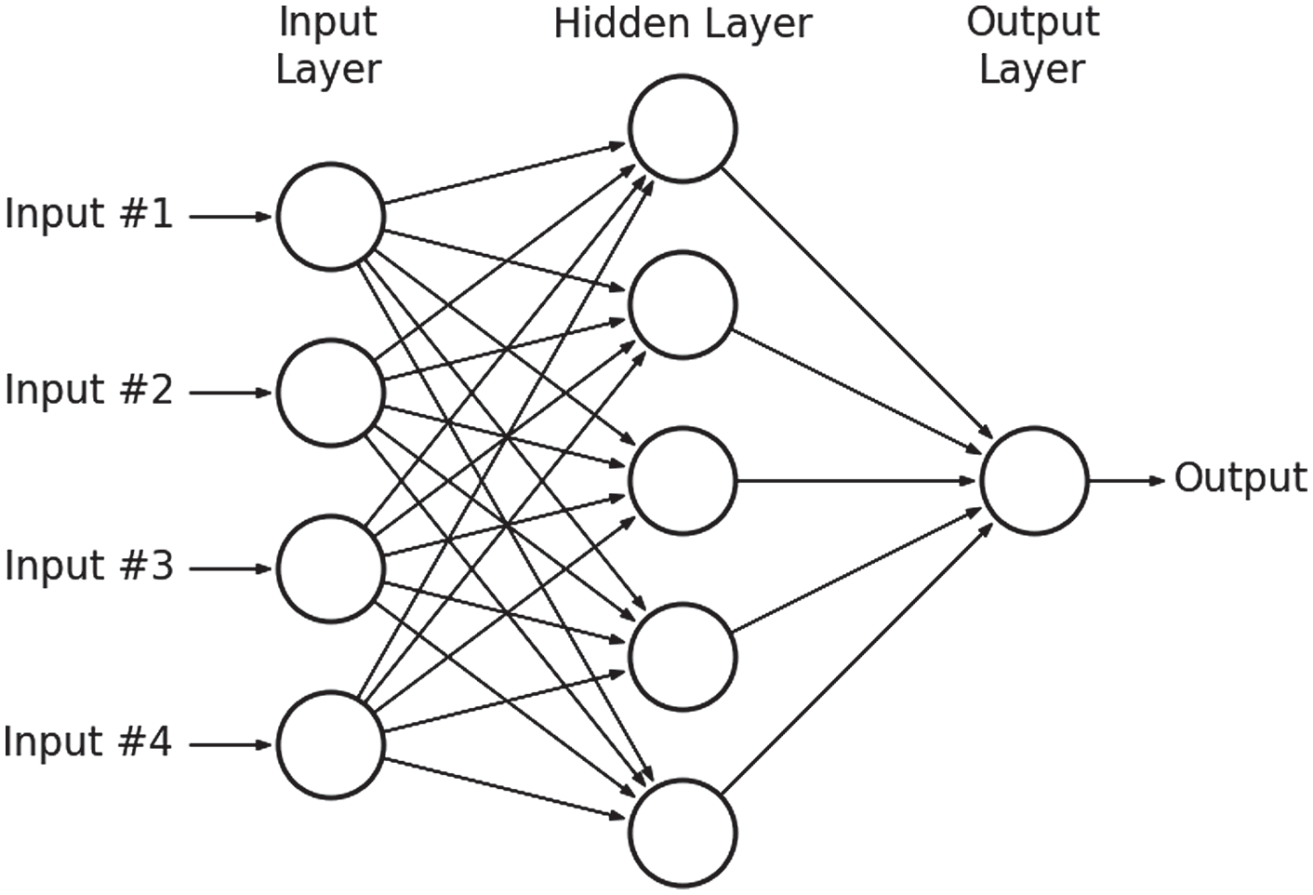
An example of a neural network framework. Circles represent individual equations which are fed data from all connected nodes. The lack of a 1-1 ratio of nodes in each layer of the network forces the model to condense information and leads to the most important information being determined iteratively through backpropagation of error (Ivezic et al., 2014).

Neural networks, or artificial neural networks, are actually a combination of many algorithms in a network, where layers of nodes, representing algorithms, feed outputs from the previous layer of nodes as inputs to the next layer, until the final layer’s output is used as the prediction (McCulloch and Pitts, 1943). Figure 5 shows a typical framework for neural network, with raw information being fed into the left and predictions coming from the right. Nodes each represent a nondescript function, typically those that make small changes to inputs, allowing for better control at each node over the final prediction. The real power for a problem with the complexity of individualized feeding is the idea of backpropagation, where the accuracy of prediction is back-propagated through the nodes of a network to re-weight the importance of each node, thereby ensuring better accuracy on the same example datum if presented again (Werbos, 1974). Put simply, backpropagation allows us to distribute error through the existing network. Neural networks have been shown to detect patterns in highly nonlinear data, which is nearly impossible for linear models (Fukushima, 1980).

Reinforcement learning is another key concept in the field of machine learning and is crucial for problems where cost functions are not explicit, like in predicting feed intake. That is, we do not know the exact cost of overfeeding or underfeeding. Suppose we are training a model to tell a farmer how to feed each cow, but the farmer is well-informed and keeps adjusting the predictions. If we were trying to minimize the need for farmer intervention, our feedback loop would weight errors based on the farmer’s adjustment to each prediction. That is to say the recurrent neural network is estimating the model that limits error under the unknown cost function. The framework starts with substantial uncertainty about the cost function and the network performs poorly; then, the network is trained and the model parameterized to decrease the cumulative costs. This is done in an updating manner called a Markov decision process (Howard, 1960). In the real world, our farmers are likely not omniscient, but the ability to estimate models under cost uncertainty can still be utilized to choose better models for actual decision making, because the cost of feeding decisions is not fixed or known, but predictions must be made every day for every cow. In fact, reinforcement models are seen in many places where decisions must be made, despite uncertainty about their costs, like game-playing algorithms and resource allocation problems (Damas et al., 2000).

Having to make predictions faced with sometimes vast uncertainty can make prediction modeling more difficult and is surely a reason why reliable levels III and IV cyberphysical systems are not seen in animal agriculture. For example, a model built to predict appropriate plane ticket costs will have a large amount of training data, because there have been many flights before. But how will a model predict the appropriate desire for a plane ticket in the days after a terrorist attack? This is a mainstream example, but consider one in the context of feeding animals. Assume a scenario where predictions for a cow’s intake have been very accurate, then she gets her foot caught in the parlor and is in a great deal of pain, the injury is not caught immediately and will not be fed into the model as an explicit variable. Is it correct to punish the model for incorrectly predicting intake on this day? Likely not, because a known, but unanticipated, event can explain the variation. This example points to a major challenge with deploying these modeling techniques on-farm. If allowed to iterate and update in an unrestricted manner, the model will try to assign weights to other factors to explain why the cow reduced intake the day she injured herself. For example, if activity data were included in the model, the weight on activity responses might be updated because we would anticipate activity to also change with the injured hoof. However, the model may take some time to recover from this prediction to correct the weight on activity under a noninjured scenario, resulting in a period of time where predictions were poor. A solution to this type of challenge would be to include an injury variable in the model to account for these types of cases; however, the point of the example is that there is always opportunity for factors exogenous to the model to influence the behavior of the response variable. When building and deploying these analytics, we must consider that reality. Another solution to the challenge is to omit data from the day in question. However, that opportunity introduces the issue of human perception with respect to identifying exogenous causes and correctly differentiating them from endogenous causes. It is important to keep in mind that we cannot leave out predictions that are not correct without reason, because every cow needs to get a prediction every day. A different solution might be found in the training of the model. Instead of focusing on minimizing the average cost of a prediction, it is possible to train the model on minimizing the maximum cost of prediction. The measure of costs relates to a secondary problem plaguing models of all varieties today: how to choose the cost functions, or, how to know which model is best.

### Challenges with model selection and evaluation

There are a number of model evaluation statistics used commonly to assess the precision and accuracy of predictions; however, when models are applied as analytics in conjunction with sensors and in the context of cyberphysical systems, the system as a whole is often evaluated on the basis of sensitivity and specificity. Indeed, in an example outside nutrition, there are actually International Standards Organization standards for sensitivity and specificity for cyberphysical systems formulated initially for automated detection of mastitis (Rutten et al., 2013). Sensitivity is a model’s ability to detect positive cases, that is, the percentage of all true positives that are detected. Specificity is the same metric applied to negative cases, namely the percentage of total negative cases that the model detects correctly. High specificity and low sensitivity leads to models that rarely detect (predict) a positive case, while the opposite would be true of high sensitivity, low specificity models. If detecting metabolic disease is an important attribute of the precision feeding system, a positive case might be an animal with metabolic disease whereas a negative case might be an animal free from disease. Although both of these calculations are extremely important for a useful cyberphysical system model in animal agriculture, false alarms can become an issue, especially in cases where the proportion of positive to negative cases is skewed in the overall population. In the case of models that detect animal conditions to alert farmers, the positive predictive value is a third measure of model accuracy that should be considered. The positive predictive value can be thought of as the probability that an alert (predicted positive case) actually is positive. Models with low positive predictive value will have more false alarms. Although positive predictive value would not be useful in the proportion of positive to negative cases in the population was equal, in many disease detection, less than 1% of cow days on a typical farm will be positive.

When we consider the example of predicting intake, or designing an ideal supplementation strategy for a cow, the use of sensitivity and specificity for model evaluation becomes more nebulous. Undoubtedly, it is more important to know by how much you over- or under-predicted a response like intake or milk yield than it is to know the binary directionality of the residual. A number of statistics (root mean squared error, mean absolute error, etc.) are available to quantify fit in this manner. However, as discussed above, when making recommendations on-farm, incorporating the cost of these decisions is perhaps most important. Working more explicitly to tie performance predictions to economic data on-farm will be an important step in advancing analytics of precision feeding.

### When are the analytics good enough?

As John von Neumann said, “truth … is much too complicated to allow anything but approximations” (Szász, 2011). Approximations are a necessary evil, particularly in the business of feeding animals. Livestock nutrition is a complex science, verging on an art form, and successful nutritionists combine analytics and exogenous information to optimize productivity of their farms. A cyberphysical system, almost by design, limits the opportunity for exogenous data, or at a minimum, changes the way that exogenous data will influence the system. To assess gold standards for when a cyberphysical system is good enough for deployment to farms, it may be useful to evaluate the standards professional nutritionists use for making feeding recommendations. Many nutritionists have a dollar value or a milk response cutoff that they believe a product, or feeding recommendation, must be expected to achieve before it should be recommended to a farmer. Gaining consensus on those cutoffs may be one way to evaluate the relevance of precision nutrition analytics from an industry context. Although it is possible to set more objective cutoffs, creating such an objective cutoff implies that a given model’s knowledge completely covers that of the experts, which is very unlikely. Although models can help weigh options in complex environments, they are only as complex as the data they are trained on, and thus by default are less informed than an expert who has the opportunity to see exogenous and endogenous variables. Further work is needed to identify the best strategies to combine and incorporating expert opinion/knowledge into a cyberphysical system focused on animal feeding.

## About the Authors

Douglas M. Liebe received his BS in Animal Sciences from the Ohio State University. He is currently in a PhD position at Virginia Tech focused on the role of data analytics for making management decisions in agriculture. Liebe’s previous work involved mathematical modeling and sustainability in animal production systems.

Robin R. White obtained a BS and PhD in Animal Sciences from Washington State University. Her doctoral work focused on mathematical modeling of sustainable beef production systems. White currently runs a three-tiered research program at Virginia Tech with basic research focused on optimizing rumen fermentation, applied research developing analytics for enhanced feed efficiency, and systems-oriented research focused on describing sustainability of livestock production systems.
